# Reaction Pathways Involved in CH_4_ Conversion on Pd/Al_2_O_3_ Catalysts: TAP as a Powerful Tool for the Elucidation of the Effective Role of the Metal/Support Interface

**DOI:** 10.3389/fchem.2016.00007

**Published:** 2016-02-16

**Authors:** Y. Renème, S. Pietrzyk, F. Dhainaut, M. Chaar, A. C. van Veen, Pascal Granger

**Affiliations:** ^1^Unité de Catalyse et de Chimie du Solide, UMR Centre National de la Recherche Scientifique 8181, Université Lille 1 - Sciences et TechnologiesVilleneuve d'Ascq, France; ^2^Ecole Nationale Supérieure de Chimie de LilleVilleneuve d'Ascq, France; ^3^Laboratory of Industrial Chemistry, Ruhr-University BochumBochum, Germany

**Keywords:** CH_4_ adsorption, palladium, TAP reactor, metal/support interface, NGV catalyst

## Abstract

Temporal Analysis of Products (TAP) investigation on Natural Gas-fueled Vehicle (NGV) catalysts provides information related to the nature of reaction steps involved over noble metals and at the metal-support interface. The determination of accurate kinetic parameters for methane adsorption from single pulse experiments and subsequent investigation of sequential surface reactions from alternative CH_4_/O_2_ pulse experiments is the first step toward the establishment of relevant structure/activity relationships which can highlight the importance of the metal/support interface on freshly-prepared and aged single palladium based catalysts.

## Introduction and scientific background

Temporal Analysis of Products (TAP) reactors allow qualitative and quantitative characterization of the kinetics of elementary steps such as adsorption, surface reactions, and diffusion processes on a wide variety of catalysts. Particular attention in the past two decades was focused on the understanding of surface processes over supported polycristalline catalysts (Gleaves et al., 1988, 2010; Gleaves and Harkins, 1991; Yablonsky et al., 2003). Indeed, pulse TAP experiments performed under ultra-high-vacuum (UHV) come near to surface science studies with the advantage to investigate industrial heterogeneous catalysts rather than model surfaces such as single crystals. Experimental conditions drastically differ from those currently encountered during classical steady-state kinetic experiments at atmospheric pressure and/or in realistic operating conditions of industrial catalytic processes. Indeed, a significant pressure gap has to be taken into account. However, prominent observations on the surface chemistry can be obtained from the injection of small amount of reactants corresponding to extremely low coverages. The suppression of the influence of diffusion on observed reaction rates, the derivation of simple laws of Knudsen domain that account for the diffusion in bed void spaces, and considering the surface characteristics as constant in typical experiments (single-pulse TAP experiments), make possible the determination of kinetic constants of elementary steps with characteristic times of order of milliseconds and, in so-called Thin-Zone TAP reactor, to determine directly reaction rates without prior assumptions on the nature of reaction mechanisms. Small amounts of reacting gases, of the order of 1 nmol, used in such experiments, suppress the influence of thermal effects of reactions studied (Perez-Ramirez et al., 2004), which may distort the results in classical kinetic experiments (Dissanayake et al., 1993). In this sense, the strategies associated with the development of TAP reactors offer an alternative to classical kinetic studies and surface science approaches (Kondratenko and Pérez-Ramírez, 2006; Perez-Ramirez and Kondratenko, 2007; Gleaves et al., 2010).

TAP reactor can be useful for depicting surface processes on nanosized noble metal particles dispersed on conventional alumina supports as the most representative porous materials for industrial applications. Kinetic parameters obtained by modeling the temporal responses can provide arguments for further discussion on the nature of steps involved over active sites, only composed of noble metal atoms, or involving the metal-support interface. Conventionally, Thermo-Programmed Desorption of hydrogen pre-adsorbed on alumina support and desorbing after reverse spillover to metal (Kramer and Andre, 1979) or by isotopic exchange experiments led to significant information (Duprez, 1997, 2006; Benkhaled et al., 2008), from which kinetic models for H_2_ exchange between metal and support and its diffusion on the support were established (Kramer and Andre, 1979; Benkhaled et al., 2008).

In TAP experiments a direct influence of the support has been suggested to explain the absence of water peak during CH_4_ oxidation on alumina-supported Rh and Ru catalysts, and the behavior of the baselines. However, these information are often qualitative (Wang et al., 1996, 2003; Schuurman et al., 1998; O'Connor et al., 2006; Perez-Ramirez and Kondratenko, 2007) despite this technique can provide accurate kinetic parameters which can reflect the structure sensitivity of surface processes or characterize the participation of the metal/support interface.

Up to now, small model molecules have been extensively studied producing a narrow panel of products that can be correctly analyzed by Quadrupolar Mass Spectrometry (QMS) limiting the usual interferences on the m/z values. By way of illustration, methane adsorption has been preferentially investigated over Ni and more recently on Pd based catalysts with the emergence of Natural Gas fueled Vehicle catalysts (NGV) but finally leading to a complex surface chemistry with sometimes controversial assessments as illustrated in Scheme 1. As shown various parallel pathways reveal the importance of oxygen coverage in the hydrogen abstraction from gaseous CH_4_ or chemisorbed CH_x_ species (Wang et al., 1996, 2003; Schuurman et al., 1998; O'Connor et al., 2006) and also point out the experimental fact that OH groups would be a more efficient oxidizer than chemisorbed O atoms (Maestri et al., 2008). The emergence of theoretical calculations based of the Density Functional Theory (DFT) provided outstanding information but essentially on model surfaces (Abild-Pedersen et al., 2005; Bunnik and Kramer, 2006) that allows the clarification of the mechanism of CH_4_ oxidation with a lowest activation barrier on Pd_x_Ce_1−x_O_2_(111) (Mayernick and Janik, 2011) compared to Pd(111) and pre-covered PdO(111). Such deviation can be related to the enhancement of OSC properties of ceria particularly when noble metals closely interact with ceria. In the particular case of alumina, it is well-recognized that Al_2_O_3_ can act as oxygen reservoir. Oxygen species needed to activate methane on noble metals can be supplied through spillover process of OH groups (Wang et al., 1996, 2003; Hofstad et al., 1998; Schuurman et al., 1998).

**Scheme 1:** **Representing steps involved during methane activation over noble metal based catalysts depending on the redox pretreatment**.

**On pre-reduced Pd/Al_2_O_3_**				**On pre-oxidized Pd/Al_2_O_3_**			
CH_4_ + 2S_1_	→	CH_3, ads_ + H_ads_	(1)	CH_4_ + O_ads_	→	CH_3, ads_ + OH_ads_	(9)
CH_3, ads_ + S_1_	→	CH_2, ads_ + H_ads_	(2)	CH_3, ads_ + O_ads_	→	CH_2, ads_ + OH_ads_	(10)
CH_2, ads_ + S_1_	→	CH_ads_ + H_ads_	(3)	CH_2, ads_ + O_ads_	→	CH_ads_ + H_ads_	(11)
CH_ads_ + S_1_	→	C_ads_ + H_ads_	(4)	CH_ads_ + O_ads_	→	C_ads_ + OH_ads_	(12)
**OH_s_ + S_1_**	⇆	**OH_ads_ + S_2_**	**(5)**	**OH_s_ + S_1_**	⇆	**OH_ads_ + S_2_**	**(5)**
C_ads_ + OH_ads_	→	CO_ads_ + H_ads_	(6)	C_ads_ + OH_ads_	→	CO_ads_ + H_ads_	(6)
CO_ads_	⇆	CO + S_1_	(7)	CO_ads_ + O_ads_	→	CO_2_ + 2S_1_	(13)
2H_ads_	⇆	H_2_ + 2S_1_	(8)	OH_ads_ + H_ads_	→	H_2_O + 2S_1_	(14)

*S_1_ represents adsorption site on the metal and S_2_ on the alumina support. OH_s_ stands for OH species adsorbed on Al_2_O_3_. Steps involving the support are in bold*.

Up to now, controversial explanations can be found in the literature regarding the effective role of oxygen to activate methane on Pd based catalysts. Sometimes beneficial effects are reported (Wang and Liu, 2006) but in contradiction with Valden et al. (1996, 1997) who observed from UHV mass spectrometry analysis the reverse trend on Pd(110) and Pd(111). These authors found a dissociative adsorption of methane strongly inhibited with an increase of the oxygen coverage. In such conditions, the probability for finding two nearest vacant sites would become very low. Even if previous investigations demonstrate the relevance of DFT calculations coupled with UHV investigations on model catalysts, their impact can be limited due to surface reconstructions under working conditions which cannot be properly predicted from theoretical calculations (Enger et al., 2008). For illustration, complex interactions take place when Pd interacts with oxygen as described elsewhere (Wolf et al., 2003) by using a model that distinguish three different chemical environments for palladium with chemisorbed oxygen on metallic palladium, surface palladium oxide, and sub-surface palladium species. Time-resolved *in situ* XANES spectroscopy and DFT modeling led to the conclusion that an intermediate O/Pt ratio would lead to an optimal activity, where an oxygen-rich surface would prevent the dissociative adsorption of methane (Becker et al., 2007). In this context, TAP analysis is a powerful technique to discriminate among different pathways which one will be kinetically significant based on the calculation of rate constants and activation barriers. This paper offers a quantitative view of the real impact of the metal/support interface based on a precise determination of kinetic constants from modeling SP experiments taking into account the peculiar porous properties of the materials. In the second part alternative pulse (AP) experiments will provide some illustrations related to surface reactivity of adsorbates over metallic sites and will emphasize the suppression of the role of the metal/support interface on the kinetics of surface processes on the aged catalysts.

## Experimental

Model Pd/Al_2_O_3_ NGV catalysts were supplied by Umicore containing 2.5 wt.% Pd. Surface characterization were reported elsewhere (Renème et al., 2012).

The TAP-1 instrument utilized for such investigation was made of two individual high speed pulse valves allowing pretreatments up to atmospheric pressure prior to pulse experiments employing the so-called high pressure assembly (van Veen et al., 2002). A stainless steel microreactor was used with a quartz insert (van Veen et al., 2001). During single pulse experiments (SP), the m/z signals at 40 (Ar), 20 (Ne, Ar^2+^), 15 (CH_4_), 32 (O_2_), 44 (CO_2_), 28 (CO, CO_2_, N_2_), 2 (H_2_, CH_4_), and 18 (H_2_O) were recorded. The calibration was performed through separate experiments by using CH_4_-Ar, CO_2_-Ar, and O_2_-Ne mixtures, with the micro-reactor filled with quartz wool and silica inert only. Prior to SP and AP experiments, pre-reductive thermal treatments were achieved under a continuous flow of 72 mbar H_2_ for 30 min at 450°C while pre-oxidation was performed under flowing O_2_ at for 50 min at the same temperature.

## Results and discussion

### Single methane pulse experiments

#### Temporal response analysis

**Figure 1:**
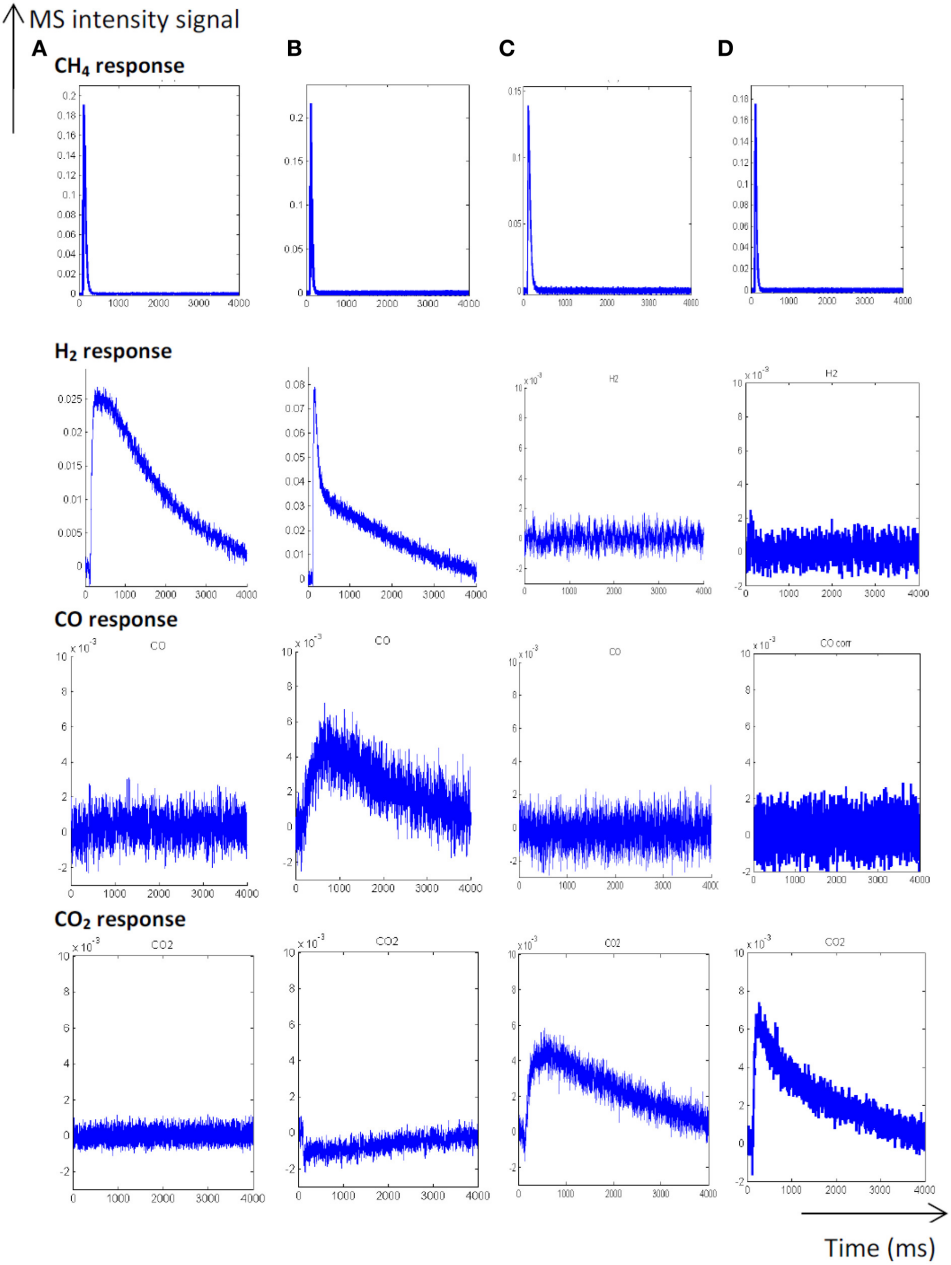
**Temporal responses from single TAP experiments at 400°C (A) and 550°C (B) on fresh-2.5 wt.% Pd/Al_**2**_O_**3**_ pre-reduced at 550°C**. Temporal responses recorded at 400°C **(C)** and 550°C **(D)** on pre-activated samples in pure oxygen at 450°C [Reproduced with permission from reference Granger and Pietrzyk (2014)].

**Figure 2:**
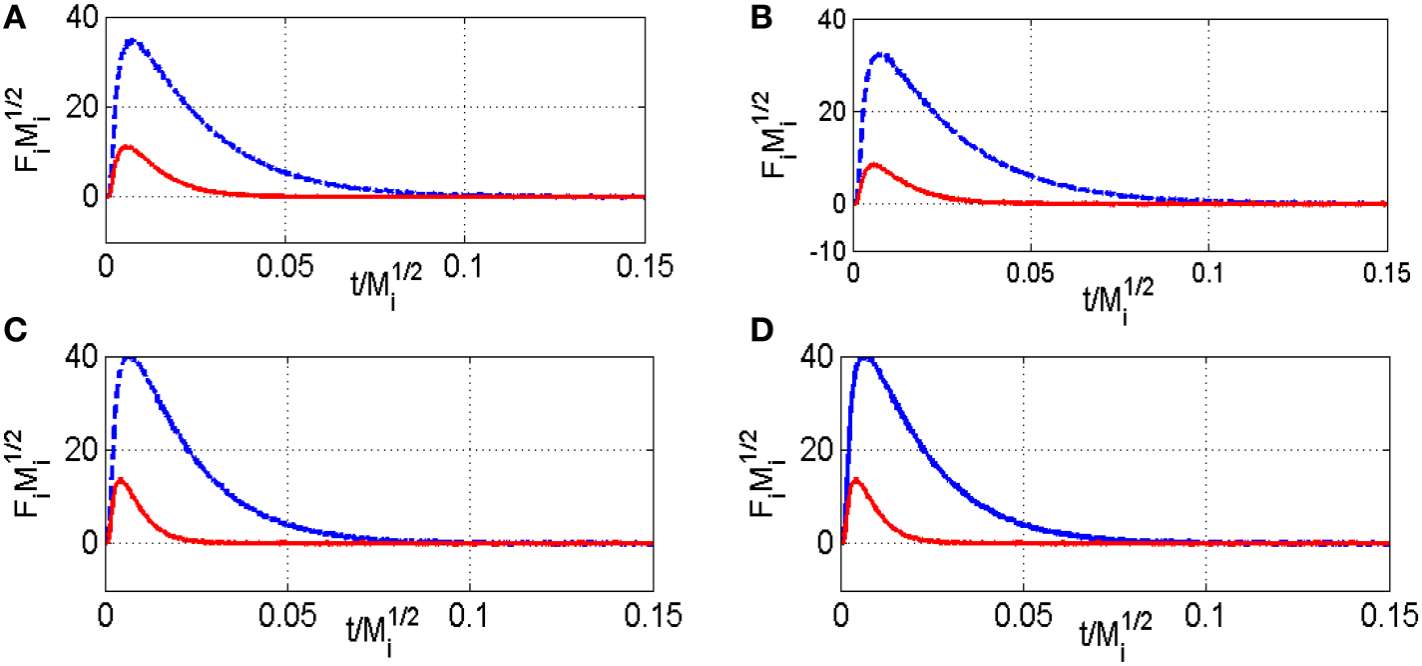
**Comparison of the normalized CH_**4**_ (red) and Ar (blue) flow curves F_**i**_${\text{M}}_{\text{i}}^{1∕2}$ vs. t/${\text{M}}_{\text{i}}^{1∕2}$ at 400°C (A) and 550°C (C) on fresh-2.5 wt.Pd/Al_**2**_O_**3**_ pre-reduced in 72 mbar H_**2**_ at 550°C for 30 min, and at 400°C (B) and 550°C (D) on pre-activated samples in pure oxygen at 450°C for 50 min—F_**i**_ stands for the pulse intensity normalized multiplied by M_**i**_ the molar weight of the compound I–Ar curve in blue and methane curve in red**.

As earlier discussed in Figure 1, different distribution products were recorded depending on the composition of the pre-covered surfaces (Granger and Pietrzyk, 2014). On reduced Pd/Al_2_O_3_, CO, and H_2_ mainly form, whereas the production of CO_2_ predominates on an oxygen pre-covered surface. The absence of signal at m/z = 18 can be explained by a very slow desorption of water and/or by a fast re-adsorption on the metal and/or the alumina support in agreement with earlier statements on γ-alumina-supported Rh (Wang et al., 1998) and Ru catalysts (Wang et al., 2003). On the other hand, such processes have not been reported on unsupported or supported Rh based catalysts on α-alumina (Hofstad et al., 1998). The comparison of the outlet flow curves of H_2_ and CO on reduced samples provides different information: (i) First, a broad signal appears at 400°C on the fresh catalyst and then (ii) another sharp signal arises with an increase in temperature, revealing the existence of two parallel pathways for H_2_ production. The initial fast process is not accompanied with a parallel CO formation that underlines the predominant CH_4_ decomposition to gaseous H_2_ and chemisorbed carbidic species (C_ads_) on Pd active centers described in Scheme 1. Afterwards, a slow H_2_ production takes place accompanied with a slow production of CO. Similar trends are still observable from methane SP experiments performed at 550°C. As exemplified a faster decomposition of methane to carbidic species occurs accompanied with a subsequent slower H_2_ production coupled to a more accentuated CO formation. On an oxygen pre-adsorbed surface, the absence of H_2_ formation at 400 and 550°C is likely related to the involvement of fast reaction with chemisorbed oxygen atoms from the dissociative adsorption of O_2_ onto metallic Pd species. As expected, CO_2_ predominantly forms. Normalized molar flow rates of methane in Figure 2, have been calculated by using the same methodology earlier described for a six-zone microreactor (Gleaves et al., 1997). As observed, no significant impact of the thermal pretreatment is noticeable either in oxidative or reductive conditions on fresh catalysts at the two selected temperature. Further comparisons with the reference outlet Ar signal agrees with a direct dissociation mechanism rather than a adsorption mechanism involving the intermediate formation of a molecular precursor state as described elsewhere on Pt (111), Pt (110) and Pd (110) (Valden et al., 1996, 1997). Such an explanation seems in rather good agreement with a large conversion of methane (>84%) once chemisorbed (see Table 1).

**Table 1:** **Surface characterization of freshly-prepared and aged supported Pd catalyst and related diffusion and kinetics constant from modeling single pulse TAP experiments**.

**Catalysts**	**Dispersion^a^(%)**	**d*_*Pd*_*^b^(nm)**	**Pretreatment**	**SP Reaction Temp**.		**${\text{D}}_{\text{b}}^{{}^{\circ}}$/ϵ_b_**	**${\text{D}}_{p}^{{}^{\circ}}$/ϵ_p_**	**${\text{k}}_{\text{ads}}^{\text{c}}$**	**E (kJ.mol^−1^)**
				**400°C**	**550°C**				
2.5 wt.% Pd/Al_2_O_3_	0.26	4.3	Reduced	0.84	0.86	6.2 × 10^−5^	1.4 × 10^−10^	0.48	7.0
			Oxidized	0.88	0.89	5.8 × 10^−5^	1.4 × 10^−10^	0.55	5.0
Aged-2.5 wt.% Pd /Al_2_O_3_^d^	0.13	8.8	Reduced	0.01	0.21	6.7 × 10^−5^	1.4 × 10^−10^	0.01	45.0
			Oxidized	0.58	0.84	6.5 × 10^−5^	1.4 × 10^−10^	0.41	27.0

a *Calculated from H_2_ titration at 100°C assuming atomic H/Pd = 1*.

b *estimated from the Pd dispersion assuming hemispherical particle*.

*^c^Calculated at 417°C expressed in ${\text{m}}_{gas}^{3}$ s^−1^${\text{kg}}_{cata}^{-1}$*.

d *After exposure to 10 vol.% H_2_O diluted in air at 980°C for 4 h*.

**Figure 3:**
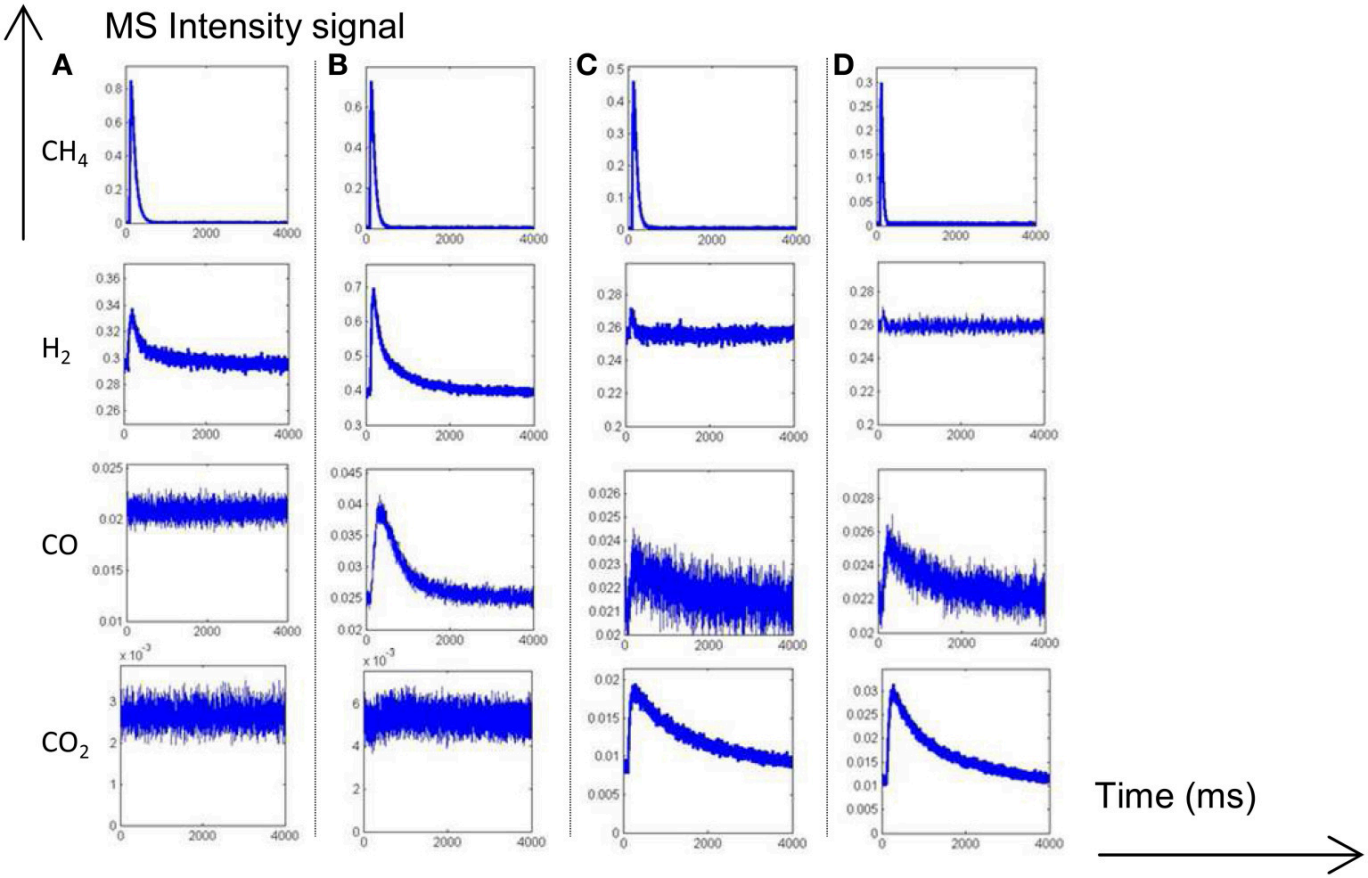
**MS responses during single TAP experiments at 400°C (A) and 550°C (B) on aged-2.5 wt.% Pd/Al_**2**_O_**3**_ pre-reduced in 72 mbar H_2_ at 550°C for 30 min**. MS responses recorded at 400°C **(C)** and 550°C **(D)** on pre-activated samples in pure oxygen at 450°C for 50 min. Corrected data from QMS tuned to m/z = 15 (CH_4_), 44 (CO_2_), 28 (CO, CO_2_), and 2 (H_2_, CH_4_).

**Figure 4:**
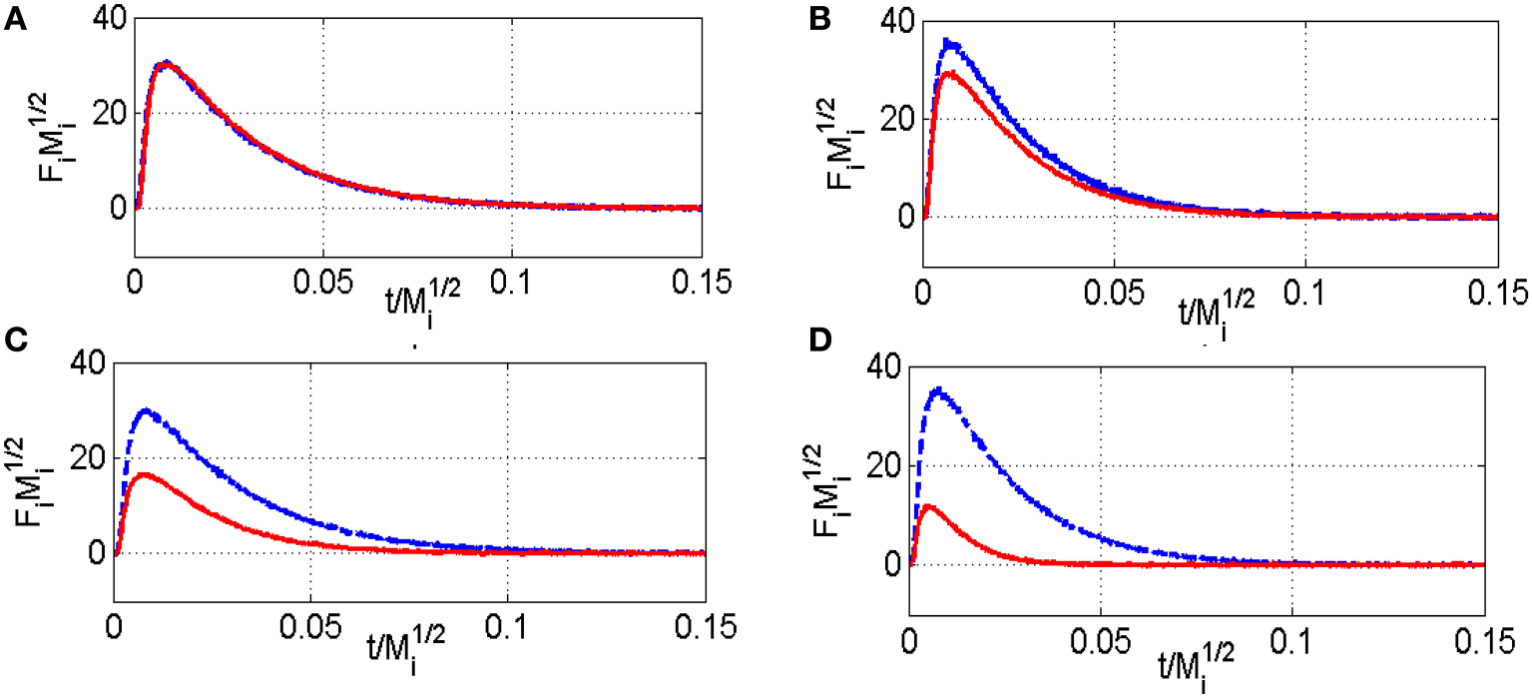
**Comparison of the normalized CH_**4**_ (red) and Ar (blue) flow curves F_**i**_${\text{M}}_{\text{i}}^{1∕2}$ vs. t/${\text{M}}_{\text{i}}^{1∕2}$ recorded at 400°C (A) and 550°C (C) on aged-2.5 wt.Pd/Al_**2**_O_**3**_ pre-reduced in 72 mbar H_**2**_ at 550°C for 30 min and at 400°C (B) and 500°C (D) on pre-activated samples in pure oxygen at 450°C for 50 min—F_**i**_ stands for the pulse intensity normalized multiplied by M_**i**_ the molar weight of the compound i**.

Thermal aging in wet atmosphere (10 vol.% H_2_O diluted in air) was implemented to speed up surface reconstructions that usually occur on NGV catalysts after long term running conditions. These reconstructions are essentially associated to particle sintering which can drastically alter the coordination of surface atoms accessible to gaseous reactants. In addition, an alteration of the metal-support interface and a loss of the textural properties can occur and modify the rate of diffusion processes. All these processes may influence the rate of methane reforming (Ligthard et al., 2011). Interestingly, the examination of the temporal MS responses in Figure 3 shows the disappearance of the simultaneous slow H_2_ and CO production processes. As a consequence, the extremely low conversion of methane recorded at 400°C on aged catalysts can be easily explained by changes in morphology and in the extent of the metal/support interface (see Table 1). Such a trend is also evidenced from SP experiments at 550°C even if a slight increase in conversion is discernible. Normalized outlet flow curves in Figure 4 emphasize the drastic loss of the adsorptive properties of pre-reduced aged-Pd/Al_2_O_3_. On the other hand, the behavior of the aged sample, pre-adsorbed by oxygen, strongly differs because in that case the adsorptive capacity toward methane is partly preserved at 400°C whereas aged and fresh sample seem to behave similarly at 550°C with comparable normalized flow curves (see Figures 2D, 4D) and conversions (0.89 on the fresh sample vs. 0.84 on the aged one). Based on this comparison the observed rate enhancements in methane conversion on oxygen pre-covered samples and in oxygen desorption with a rise in temperature seem in rather good agreement with previous interpretation suggesting an optimal O/Pt ratio (Becker et al., 2007) to get high conversion. Such an explanation could satisfactorily explain our results on aged Pd/Al_2_O_3_ whereas oxygen-rich surface as expected at 400°C would partly inhibit the dissociative adsorption of methane.

#### Kinetic modeling

The outlet flow rate curves for methane from SP TAP experiments were modeled using COMSOL Multiphysics, version 3.5a, and Matlab R2009b to compare predicted and experimental data. The model parameters were optimized using Matlab “*fminsearch”* function, based on the Nelder-Mead algorithm. Model for methane adsorption implemented 1-D approximation and assumed Knudsen diffusion for a gas-phase species *i*. The contribution of quartz wool or metal grids was considered in the calculations as separate solid zones. The textural properties were also taken into account in the estimation of the diffusivity constant in the temperature range of 400–550°C according to earlier model proposals (Ligthard et al., 2011) and contrarily to previous investigations neglecting the macro-mesoporous texture and further related internal diffusion limitations (Phanawadee et al., 2005). The parameters of this model are diffusivities for the inter-particle space, those for the intra-particle void space representing the pores, and the mean particle radius used to estimate the surface of particles per unit volume of the bed, and considered as equal to the pore length. Hence, two different types of voids have been considered which account for the existence of micropores characterized by the ${\text{D}}_{\text{p}}^{{}^{\circ}}$ values of the order of 2 × 10^−8^ m^2^s^−1^ with a porosity ε_p_ of about 0.2 and extra-granular voids corresponding to macropores, with ${\text{D}}_{\text{b}}^{{}^{\circ}}$ ~1 × 10^−4^ m^2^s^−1^ with a porosity ε_b_ of ~0.6. Rate constants for methane adsorption have been computed taking into account the above-mentioned restrictions due to the porous structure of the catalyst bed and efficient diffusivity coefficient in Knudsen regime collected in Table 1. Activation energies have been subsequently calculated from the slopes of Arrhenius plots on the basis of the estimates of rate constant values obtained in the temperature range 400–550°C. As seen in Table 1, rate constants on freshly-prepared and aged-Pd/Al_2_O_3_ measured at 400°C do not vary significantly likely within the margin of error except on the aged sample pre-reduced in H_2_. As earlier observed in this latter case, the adsorption capacity is quasi-completely loss at 400°C which can explain the very low calculated value. The comparison of the activation energy values reveals interesting observations. As seen in Table 1, the numerical values on fresh samples are insensitive to reductive or oxidative pre-activation thermal treatments. It is also worthwhile to note that they are much lower compared to the values calculated on the aged samples especially on the pre-reduced aged catalyst. The abnormally high values in this latter case can be partly related a very poor accuracy due to negligible estimates determined for the rate constant at low temperature. However, these overestimated values could also reflect the impact chemisorbed O atoms onto Pd particles on the methane adsorption once the metal-support is deteriorated and would not participate significantly through spillover effects of OH groups (Shuurman, 2007; Beck et al., 2009).

The unity bond index-quadratic exponential potential (UBI-QEP) method can provide straightforwardly activation barrier (Shustorovich and Sellers, 1998). Previous calculations considering the hypothesis of vacant Pd sites or pre-covered by chemisorbed O atoms according to steps (1) and (9), led to numerical solutions obtained from this method on Pd (111) of the same order of magnitude than those modeled from TAP measurements on aged Pd/Al_2_O_3_ catalysts according to the accuracy (Granger and Pietrzyk, 2014). On the other hand, some divergences arise regarding the very low experimental values determined on freshly-prepared catalysts becoming independent on the pre-activation thermal treatment. Such divergences can characterize either the involvement of different pathways for methane dissociation over Pd sites or surface diffusion processes usually characterized by low activation barriers previously found as kinetically significant on supported noble metal catalysts particularly when alumina is used as support. In fact, alumina is well-recognized as a reservoir of OH groups supplying oxygen to activate methane on metallic sites via spill-over process (Wang et al., 1996; Schuurman et al., 1998; O'Connor et al., 2006). Up to now, such a behavior has been essentially argued in the past based on the examination of the outlet flow rate for H_2_ production which demonstrates the existence of different surface processes with relative rates strongly affected by the thermal aging. The fast H_2_ production via CH_4_ decomposition highlighted in Figures 1, 3 does not seem significantly altered contrarily to the broad H_2_ response previously ascribed to OH and/or H_2_O, forms during H_2_ pulses, spilled over from the support onto the metal which strongly attenuates on aged samples. Migration and further consumption of OH groups from alumina could be consistent and kinetically significant further explaining the very low activation barrier and their insensibility to the nature of the pre-treatment under oxidizing or reducing atmosphere. Subsequent theoretical calculations support the involvement of OH species as reported elsewhere (Granger and Pietrzyk, 2014) leading to a much lower values for the activation barrier (~0 vs. 41.6 kJ.mol^−1^) when OH species assist the dissociative adsorption of methane. Hence, this comparison emphasizes the fact that OH groups from alumina could diffuse from the support to Pd sites through the metal-support and then react with adsorbed methane.

Now returning to the behavior of oxygen pre-covered fresh and aged Pd/Al_2_O_3_, it seems obvious that at low coverage chemisorbed O species can assist the dissociative methane adsorption in the course of SP experiments and would compensate the loss of Pd-Al_2_O_3_ interface due to particle sintering. This seems in agreement with methane conversion recorded at 550°C comparable on fresh and aged samples and also to a much higher activation barrier which tends to theoretical value ~47.6 kJ.mol^−1^ calculated on Pd (111) from the UBI-QEP method (Granger and Pietrzyk, 2014).

### Alternative pulse experiments on Pd/Al_2_O_3_

#### Before aging

**Figure 5:**
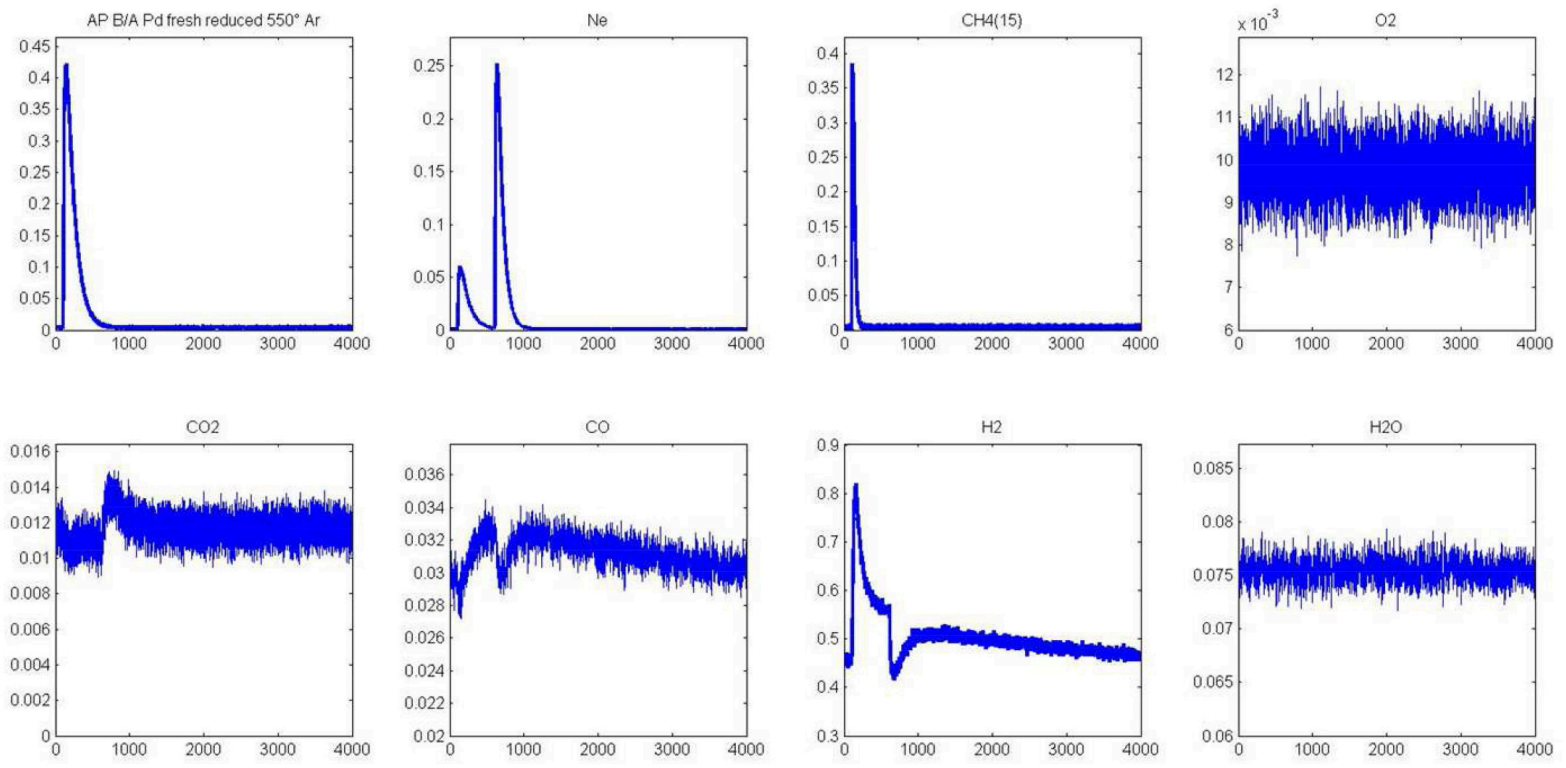
**Alternative pulses of CH_**4**_ and O_**2**_ at 550°C on fresh 2.5 wt.% Pd/Al_**2**_O_**3**_ pre-reduced in H_**2**_ at 450°C prior to AP experiments**.

**Figure 6:**
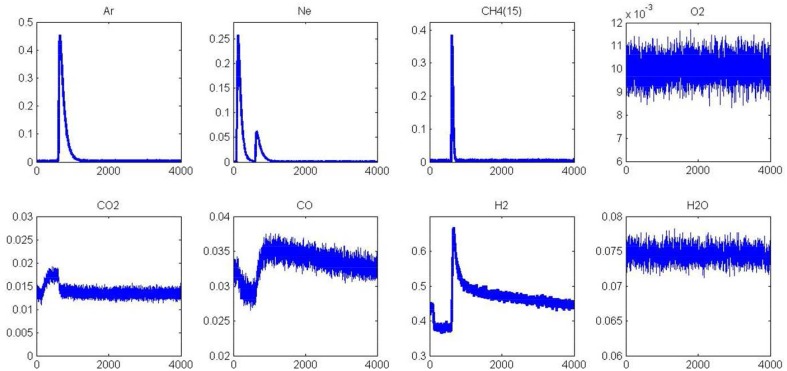
**Alternative pulse of O_**2**_ and CH_**4**_ at 550°C on fresh 2.5 wt.% Pd/Al_**2**_O_**3**_ pre-reduced in H_**2**_ at 450°C prior to AP experiments**.

**Figure 7:**
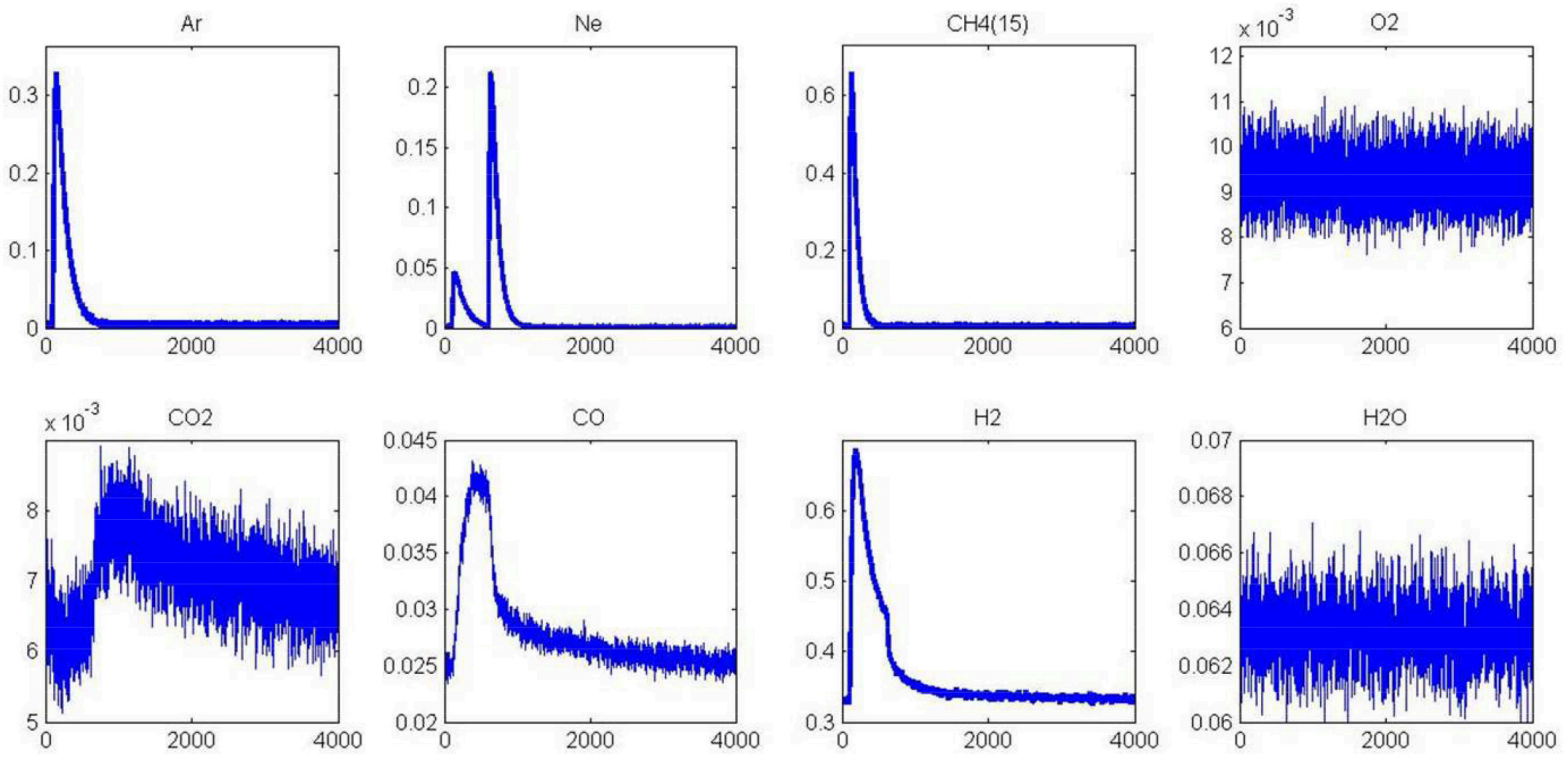
**Alternatives pulses of CH_**4**_and O_**2**_ at 550°C on aged-2.5 wt.%Pd/Al_**2**_O_**3**_ pre-reduced in H_**2**_ at 450°C prior to AP experiments**.

**Figure 8:**
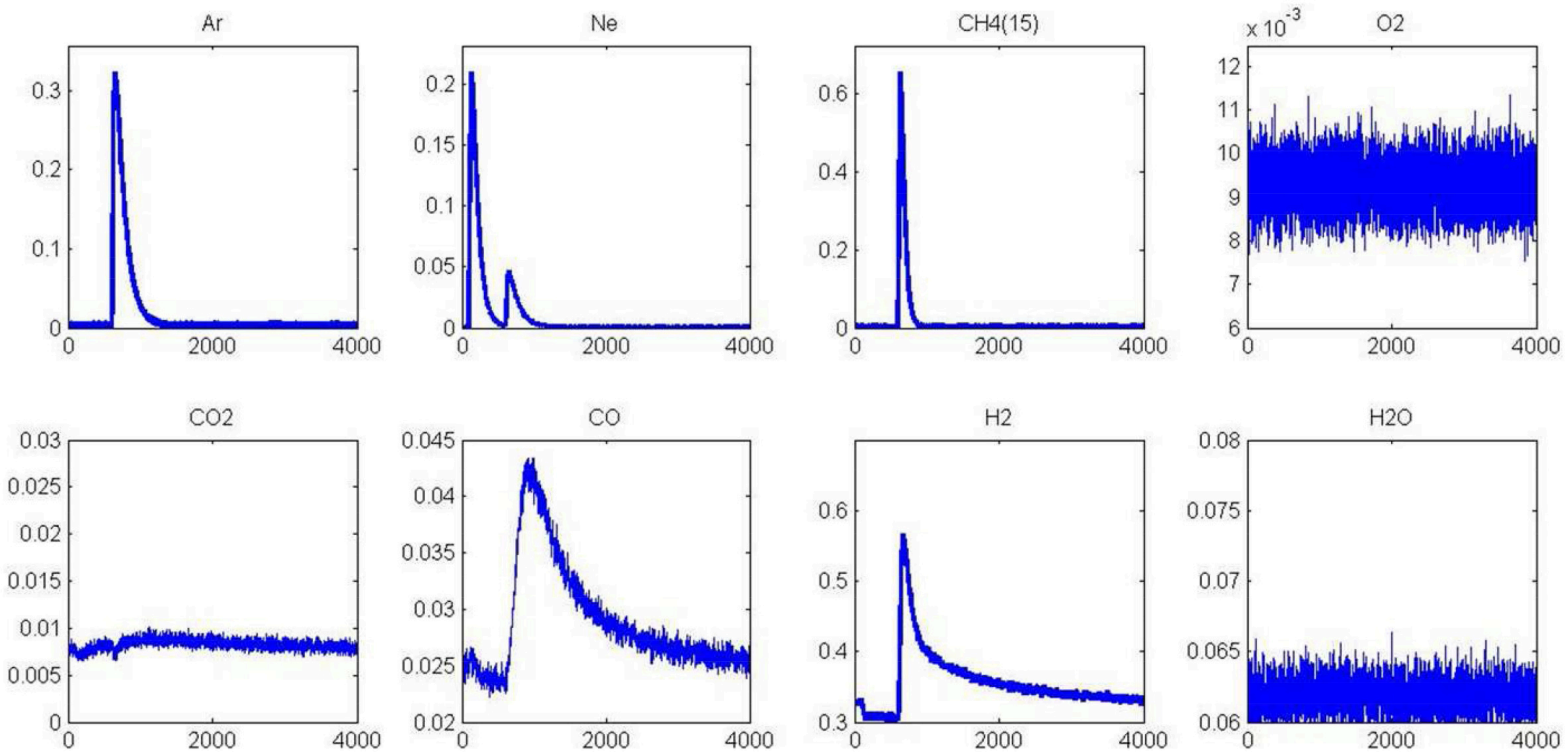
**Alternatives pulses of O_**2**_ and CH_**4**_ at 550°C on aged-2.5 wt.% Pd/Al_**2**_O_**3**_ pre-reduced in H_**2**_ at 450°C prior to AP experiments**.

Two different AP experiments are illustrated in Figures 5–**8**. In the former case, CH_4_ is initially introduced and 1 s later oxygen is pulsed. In the second experiment, the order of introduction of the reactants is reversed. It is also worthy to note that AP experiments were exclusively performed on pre-reduced catalysts after 72 mbar H_2_ exposure at 450°C for 30 min. MS responses in Figure 5 recorded at 550°C agrees with previous observations showing at the first stage of the experiment a fast production of hydrogen parallel to the decomposition of methane on metallic Pd sites. Afterwards, the H_2_ response becomes gradually broader with a tail accompanied with a simultaneous CO formation till the admission of the successive O_2_ pulse. The introduction of oxygen induces a strong disruption in H_2_ and CO responses which likely reflects the fast consumption of chemisorbed hydrogen and C-containing species on Pd by gaseous O_2_. Correlatively, a slow CO_2_ is discernible. Returning to the outlet flow curve of H_2_, the signal slowly increases once oxygen is totally consumed and this also coincides with a correlative increase of the outlet flow curve of CO similarly to previous observations prior to O_2_ admission. In the meantime, the MS response for m/z = 18 remains unchanged which suggests that water produced during surface reactions re-adsorbs and could be involved in the slow production of CO and H_2_ through spillover processes replenishing the OH groups of alumina support. Such observations would likely suggest the involvement of water and/or OH groups onto the support capable to spill-over onto the Pd and then reacting with carbidic species from methane dissociation. All those observations mimic those previously described from SP experiments in Figure 1 revealing the importance of the Pd-support interface.

A second series of AP experiments consists in injecting O_2_ prior to methane. As previously discussed, the role of oxygen remains unclear related to beneficial or inhibiting effects toward methane dissociation (Valden et al., 1996, 1997; Wang and Liu, 2006). As earlier discussed (Granger and Pietrzyk, 2014), the comparison of calculated activation barriers from the UBI-QEP method led to higher values on a pre-covered Pd (111) than on bare Pd (111) surface suggesting a detrimental effect chemisorbed O atoms. On the other hand, it was found that the dissociation of methane is clearly assisted by the presence of nearest-neighbor OH group from these theoretical calculations with much lower activation barriers. SP experiments provide more information emphasizing the beneficial effect of oxygen at very low coverage with adsorption and dissociation in the same extent on fresh and aged samples.

Returning to the examination of the MS response for oxygen in Figure 6, an extra formation of CO is observable coming from the previous AP experiment. The sharp decrease observed on the signal ascribed to CO during the first stage of the experiment is correlated to CO_2_ production. Again, a strong disruption on the CO_2_ signal is discernible, immediately after methane admission. Oxygen will preferentially react with hydrogen coming from the fast methane dissociation. Once chemisorbed oxygen atoms at the surface are totally consumed, then the long tail related to the slow production of H_2_ and CO is restored with a broader signal observed for m/z = 2 and 28 respectively.

#### After aging

As previously observed from SP experiments, thermal aging has a strong detrimental effect on methane adsorption at 400 and 550°C on a pre-reduced surface. Pre-oxidized catalysts are also significantly affected at 400°C whereas the aged and fresh samples exhibit comparable behavior at 550°C. As explained, the formation of surface oxide and/or subsurface oxygen during the oxidative pre-treatment could partly compensate the loss of the metal-support interface in this temperature conditions and then preserving the adsorptive capacity of aged-Pd/Al_2_O_3_. In order to visualize these different features toward methane adsorption, AP experiments were performed on aged catalysts pre-reduced at 450°C. Figure 7 illustrates the temporal responses obtained after successive CH_4_ and O_2_ pulses. As earlier observed, a fast H_2_ production from methane dissociation takes place. Subsequent O_2_ admission, 1 s later, still induces a strong disruption on the H_2_ signal essentially due to the preferential reaction of oxygen with hydrogen. On the other, we do not observe a restoration of H_2_ production compared to previous observations on the fresh sample in Figure 5 and this is also confirmed on the outlet flow rate of CO with the disappearance of the long tail. Hence, all these observations characterize the loss of adsorption capacity due to the alteration of the metal-support interface as previously discussed from SP experiments. Now regarding the behavior of the aged sample in Figure 8, one can notice that the H_2_ curve returns to the baseline more quickly than that observed in Figure 6 which emphasizes the fact that the degradation of the metal-support interface could slow down spillover and reverse-spillover processes.

## Conclusion

As a conclusion, this investigation illustrates the potentialities of quantitative TAP analysis for the characterization of the impact of the metal/support interface going farther on the existing studies that essentially justify the involvement of the metal/support interface based on qualitative information. Accurate determination of rate constants usually achieved from such type of studies needs accurate quantification integrating the characteristics of the different zone of the reactor including the porous structure of the materials. As demonstrated, the discussion of the order of magnitude of the rate constant would not be sufficient because the comparison of the values cannot provide decisive argument on the role of the metal-support interface. On the other hand the activation barrier reflects without any ambiguity the participation of the alumina support. Further alternative pulse experiments provide useful information which confirms the role of the Pd-alumina interface in the supply of reactive oxygen species through spillover effect.

## Author contributions

YR, PhD students who made the experiments; SP, TAP modeling response; FD, kinetics; MC, PhD students who made the experiments; AV, TAP exploitation; PG, kinetic modeling.

### Conflict of interest statement

The authors declare that the research was conducted in the absence of any commercial or financial relationships that could be construed as a potential conflict of interest.
